# Dynamics of the CD9 interactome during bacterial infection of epithelial cells by proximity labelling proteomics

**DOI:** 10.1111/febs.70291

**Published:** 2025-10-17

**Authors:** Paige A. Wolverson, Isabel Fernandes Parreira, Ruth H. Thompson, Mark O. Collins, Jonathan G. Shaw, Luke R. Green

**Affiliations:** ^1^ School of Clinical Dentistry University of Sheffield UK; ^2^ Florey Institute of Infection University of Sheffield UK; ^3^ School of Medicine and Population Health University of Sheffield UK; ^4^ Nucleic Acids Institute University of Sheffield UK; ^5^ School of Biosciences University of Sheffield UK

**Keywords:** CD9, *Neisseria meningitidis*, proximity labelling, *Staphylococcus aureus*, Tetraspanin

## Abstract

Bacterial species utilise different receptors at the cell membrane to adhere to cells. Previously, we demonstrated that interference with CD9, a human tetraspanin, reduces adherence of multiple species of bacteria to cells. CD9 is not a receptor but organises numerous commandeered host proteins at the cell membrane; however, the full interactome has not yet been delineated. Using a CD9 proximity labelling model, a first for CD9, we observed a diverse interactome, with 710 enriched proteins in uninfected cells. Proximal proteins were associated with various cellular processes, including extracellular matrix (ECM)–receptor interactions and tight junctions. Several known bacterial receptors were also detected, including CD44, CD46 and CD147. The interactome was dynamic during infection with two distinct bacterial species, *Neisseria meningitidis* and *Staphylococcus aureus*. In total, 12 human proteins were enriched during meningococcal infection, compared to one during staphylococcal infection, demonstrating different host factor requirements during CD9‐mediated bacterial adherence. CD44 or CD147 knockdown reduced staphylococcal and meningococcal adherence, respectively, but not *vice versa*. However, in combination with CD9 interference, no additive effects were observed, demonstrating association of these proteins during infection. We have developed a tool that measures changes within the CD9 interactome, demonstrated CD9 as a universal organiser of bacterial ‘adhesion platforms’, and shown efficacy of a disrupting CD9‐derived peptide.

## Graphical abstract

We describe the CD9 interactome in epithelial cells for the first time. The interactome is diverse and dynamic and changes dependent on infection with specific bacteria. We validate these data by showing functional interaction between CD9 with CD44 or CD147. We therefore demonstrate that CD9 is a universal organiser of bacterial adhesion platforms able to organise and recruit numerous host cell membrane proteins dependent on the infecting bacteria.

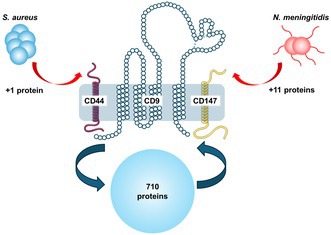

**Abbreviations**
AGC: automatic gain control
AM: acetoxymethyl
APEX: ascorbate peroxidase
BHI: brain–heart infusion
BSA: bovine serum albumin
CD: cluster of differentiation
CEACAM: carcinoembryonic antigen‐related cell adhesion molecule
cfu: colony‐forming unit
CMV: cytomegalovirus
cryo‐EM: cryogenic electron microscopy
DAPI: 4′,6‐diamidino‐2‐phenylindole
DDA: data‐dependent acquisition
EC1: small extracellular loop
EC2: large extracellular loop
ECL: enhanced chemiluminescence
ECM: extracellular matrix
EDTA: ethylenediaminetetraacetic acid
eGFP: enhanced green fluorescent protein
EGFR: epidermal growth factor receptor
EGTA: ethylene glycol tetraacetic acid
FDR: false discovery rate
FITC: fluorescein isothiocyanate
FTMS: fourier transform mass spectrometry
GAS: group A Streptococcus
GPI: glycosylphosphatidylinositol
GTP: guanosine triphosphate
HCD: higher‐energy collisional dissociation
HRP: horseradish peroxidase
HSPG: heparan sulphate proteoglycan
IgG: immunoglobulin G
IgSF3: immunoglobulin superfamily 3
IMD: invasive meningococcal disease
ITMS: ion trap mass spectrometry
KEGG: Kyoto Encyclopedia of Genes and Genomes
LB: luria broth
LC–MS/MS: liquid chromatography–tandem mass spectrometry
LFQ: label‐free quantification
MAPK: mitogen‐activated protein kinase
MOI: multiplicity of infection
NIPA: nuclear interaction partner of anaplastic lymphoma kinase
Opa: opacity protein
PBS: phosphate‐buffered saline
PCNA: proliferating cell nuclear antigen
PKC: protein kinase C
Rab: ras‐related protein in brain
Rap1: ras‐associated protein 1
RFI: relative fluorescence intensity
RIPA: radioimmunoprecipitation assay
SDS/PAGE: sodium dodecyl sulphate‐polyacrylamide electrophoresis
siRNA: small interfering RNA
SNARE: soluble NSF attachment protein receptors
TCEP: tri‐(2‐carboxyethyl) phosphine hydrochloride
TEM: tetraspanin‐enriched microdomain
TFA: trifluoroacetic acid
TM: transmembrane domain
TRIO: triple functional domain protein
WT: wild‐type

## Introduction

Tetraspanins are a superfamily of transmembrane proteins, consisting of 33 members in humans, that are classically characterised by four transmembrane domains (TM1‐TM4), a small extracellular loop (EC1), a large extracellular loop (EC2), along with short intracellular N‐ and C‐termini [1]. Sequence and structural divergence within the extracellular loops and the intracellular domains, especially within the C terminus, provides a unique function for each tetraspanin [2]. A key characteristic of tetraspanins is their ability to associate with various membrane proteins, including integrins, proteoglycans and immunoglobulin superfamily members [3, 4], to form molecular complexes known as tetraspanin‐enriched microdomains (TEMs) [5]. TEMs are involved in myriad biological processes, including cell adhesion, migration and signalling, and have been identified as a route of infection for many viral and bacterial pathogens [5, 6].

Recent studies have implicated tetraspanins in various bacterial adherence pathways, including those of pathogenic *Neisseria* spp. [7], *Staphylococcus aureus* [4, 8], *Escherichia coli* [9], *Burkholderia pseudomallei* [10, 11] and *Mycobacterium abscessus* [12]. In most cases, the tetraspanins themselves do not act as the bacterial receptors but coordinate organisation and clustering of receptors that allow for bacterial adherence, suggesting the composition of the TEM is important for initial bacterial attachment [4, 7]. For example, CD9 promotes *S. aureus* adherence to epithelial cells through organisation of the syndecans, allowing recruitment of fibronectin to create an optimal adhesive platform for the bacterium [4]. However, each bacterial species that exploits tetraspanin‐mediated adherence co‐opt different receptors during cellular invasion but the tetraspanins have been demonstrated to associate with many of these membrane proteins.

*Neisseria meningitidis*, an opportunistic bacterial pathogen able to cause invasive meningococcal disease (IMD), uses various bacterial adhesins to adhere to many different cell types [13]. The canonical adherence pathway consists of an initial interaction between type IV pili and either CD147 [14] or potentially CD46 [15]. From here, an intimate association occurs after interaction of the opacity proteins (Opa) with either heparan sulphate proteoglycans (HSPGs) or CEACAMs [16]. Similarly, *S. aureus*, a common human commensal which can also cause multiple infectious diseases including pneumonia, osteomyelitis and infective endocarditis, utilises a plethora of adhesins to associate with multiple host cell receptors [17]. Perhaps the most studied of these is the interaction of the staphylococcal fibronectin binding proteins to fibronectin which is utilised as a ‘bridge’ with α5β1 integrin [18]. Still, several other pathways have been investigated and include co‐opting host receptors such as CD36, desmoglein 1 and annexin 2 [17]. The tetraspanin CD9 has previously been demonstrated to associate with various integrins [19], HSPGs [4], immunoglobulin superfamily members [3], CD36 [20] and CD46 [21] in the formation of TEMs.

Here, we utilise proximity labelling to characterise the CD9 interactome on epithelial cells and demonstrate that different CD9‐associated TEMs are required for infection by different bacteria. We also report the dynamism of these TEMs during infection with specific proteins recruited to CD9‐associated TEMs over time. Finally, we demonstrate that a CD9‐derived peptide can disrupt these interactions and reduce specific bacterial adherence to cells. Disruption of TEMs and therefore disruption of the formation of bacterial cell surface receptor complexes is arising as a promising therapeutic target and could aid in overcoming the growing pressures associated with antimicrobial resistance.

## Results

### Disruption of CD9 can affect both meningococcal and staphylococcal adherence to epithelial cells

**Fig. 1:**
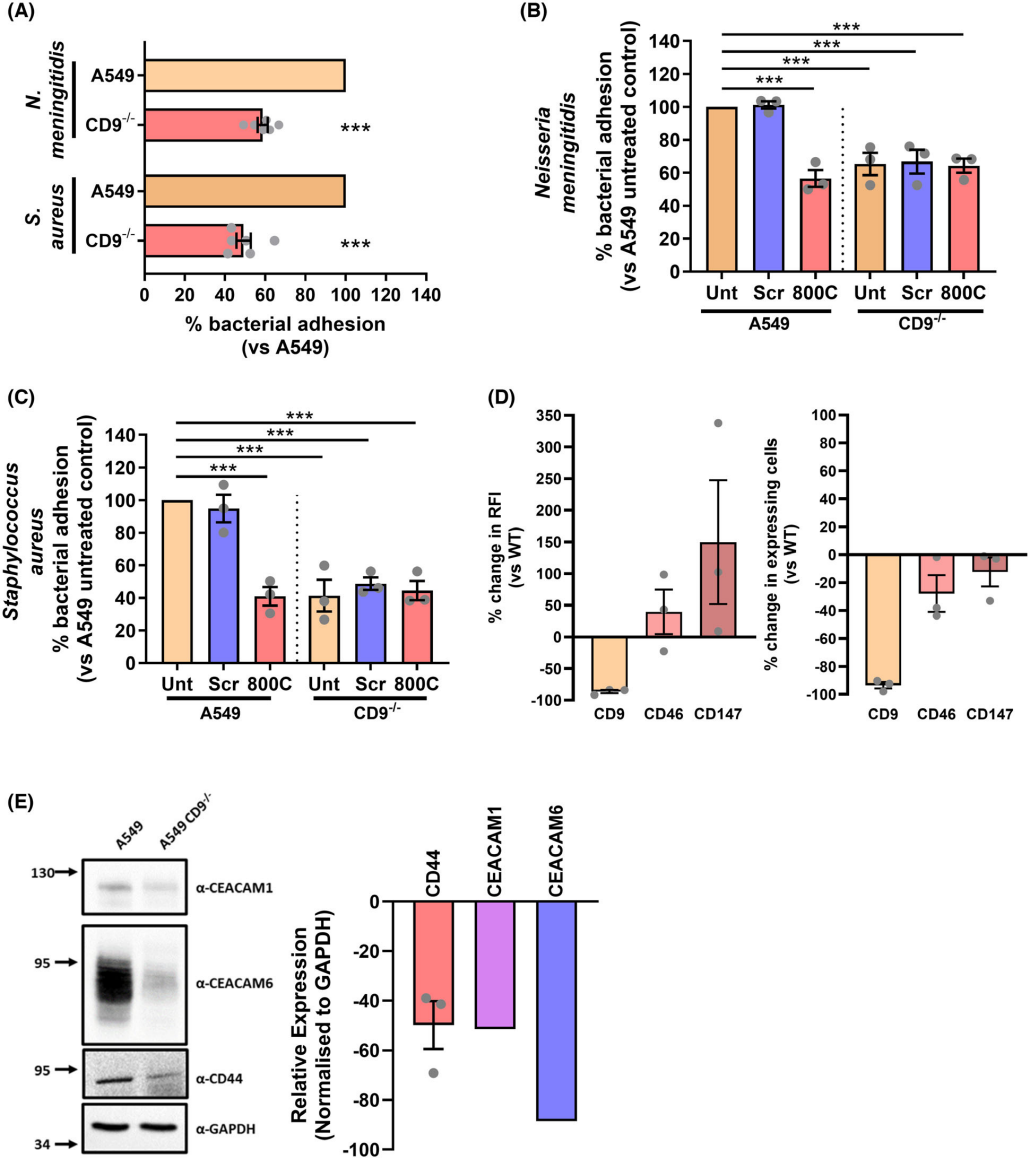
CD9 knockout affects both meningococcal and staphylococcal adherence and changes receptor expression. (A) CD9 knockout disrupts both meningococcal and staphylococcal adherence. WT and CD9^−/−^ cells were infected with either meningococci (MC58) or staphylococci (SH1000) for 60 min at a multiplicity of infection (MOI) = 50. Cells were disrupted after infection and adherent and internalised bacteria were enumerated by colony forming units (cfu). *n* = 6, mean ± SEM, one‐way ANOVA, ***P ≤ 0.001. (B, C) CD9‐derived peptide, 800C, reduces meningococcal adherence (B) and staphylococcal adherence (C) to epithelial cells. Wild‐type (WT) or CD9^−/−^ cells were pretreated with a scrambled peptide (Scr) or 800C for 60 min prior to infection. Untreated cells denoted by Unt. Cells were infected as above and adherence enumerated by cfu. *n* = 3, mean ± SEM, one‐way ANOVA, ***P ≤ 0.001. (D) Cell surface expression of meningococcal receptors measured by flow cytometry. WT or CD9^−/−^ cells were treated with an anti‐CD9 antibody (602.29), an anti‐CD46 antibody and an anti‐CD147 antibody. A mouse IgG isotype control (JC1) was included and expression was determined using a FITC‐conjugated secondary antibody. % change was calculated by comparing WT to CD9^−/−^ cells. Panels demonstrate the % change in relative fluorescence intensity (RFI) or in the number of expressing cells. *n* = 3, mean ± SEM. (E) Representative blot demonstrating expression of meningococcal and staphylococcal receptors. Whole cell lysates of WT and CD9^−/−^ cells were electrophoresed and blotted. Blots were probed with either anti‐CD44, anti‐CEACAM1 (*n* =1) or anti‐CEACAM6 (*n* = 1) antibodies. An anti‐GAPDH antibody was used as a loading control. Densitometry was calculated through imagej analysis, removing background with an empty lane and normalising to the loading control. CD44 *n* = 3, CEACAM1 and CEACAM6 *n* = 1, mean ± SEM.

Tetraspanins have previously been demonstrated to be involved in both meningococcal and staphylococcal adherence [4, 7]. These data were confirmed by measuring meningococcal and staphylococcal adherence to wild‐type (WT) A549 or tetraspanin CD9^−/−^ A549 cells. Knockout of CD9 significantly reduced both meningococcal (58.7 ± 6.1%) and staphylococcal (49.2 ± 8.7%) attachment compared to WT cells (Fig. 1A). Furthermore, a CD9‐derived peptide, 800C, can also affect both bacterial adherence pathways. Pretreatment of WT cells with 800C significantly reduced meningococcal adherence to cells compared to untreated cells (56.5 ± 8.8%; Fig. 1B). Similarly, as previously demonstrated [4], 800C treatment of WT cells significantly reduced staphylococcal adherence to cells in comparison to untreated cells (41.0 ± 9.9%; Fig. 1C). A scrambled 800C peptide control had no effect on either bacterial adherence pathway. Adherence of both bacteria was significantly reduced in CD9^−/−^ cells; however, treatment with the scrambled peptide or 800C had no further effect (Fig. 1B,C).

CD9 expression was substantially reduced in CD9^−/−^ cells in comparison to the WT cells (−86.33 ± 4.4%; Fig. 1D). Previously, we have observed that the staphylococcal receptor, syndecan‐1, is affected by removal of CD9 [4]. Here, we investigate the expression of known meningococcal receptors (CD46, CD147, CEACAM1 and CEACAM6) and a known glycoprotein implicated in staphylococcal adherence (CD44) to epithelial cells [22]. Analysis of cells by flow cytometry demonstrated that removal of CD9 increased the expression of CD46 (39.64 ± 35.0%) and CD147 (149.7 ± 97.9%) at the cell membrane; however, these increases were not significant (Fig. 1D). No significant differences were observed in the percentage of cells expressing our target proteins (Fig. 1D). Conversely, decreases were observed by western blot in CD44 expression after CD9 removal (−49.8 ± 16.8%). Expression of CEACAM1 and CEACAM6 was also reduced after CD9 removal (Fig. 1E). Thus, we have confirmed that removal of tetraspanin CD9 can affect multiple bacterial adherence pathways despite changes in expression in putative receptors.

### Efficient labelling of CD9 with TurboID

**Fig. 2:**
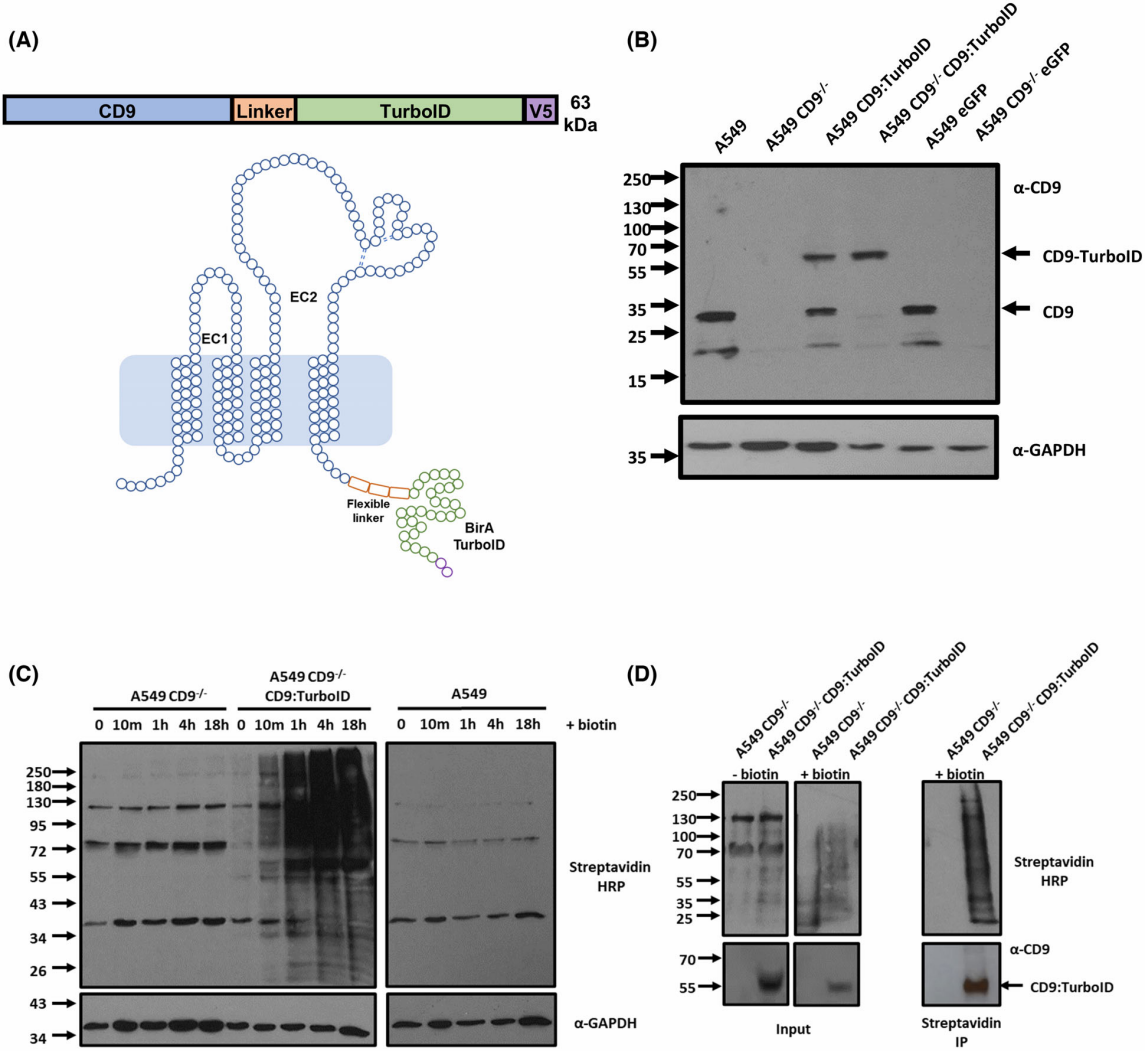
CD9:TurboID fusion construct is expressed and biotinylates proximal proteins. (A) Schematic of the CD9:TurboID fusion protein used in this study. The relative position of a flexible linker, TurboID and a V5 tag are indicated. (B) Whole cell lysates of wild‐type (WT) and CD9^−/−^ cells and those transfected with either the CD9:TurboID fusion plasmid or the eGFP control plasmid were separated by electrophoresis and blotted. Blots were probed with anti‐CD9 (MM2‐59) and anti‐GAPDH antibodies. *n* = 3. (C) Representative blot demonstrating biotinylated proteins produced by WT, CD9^−/−^ or CD9^−/−^ CD9:TurboID transfected cells. Cells were treated with 50 μm biotin for 18 h and sampled at various timepoints. Blotted proteins were probed with a streptavidin horseradish peroxidase (HRP) conjugate. *n* = 3. (D) Streptavidin bead pull‐downs demonstrate a large number of enriched biotinylated proteins. CD9^−/−^ or transfected cells were treated with 50 μm biotin for 60 min. Biotinylated proteins were pulled down from lysed cells using streptavidin beads. The input and resulting pull‐down were probed with a streptavidin HRP conjugate. *n* = 3.

We have demonstrated that CD9 interference can affect very distinct bacterial adherence pathways. We have also previously shown that CD9 does not appear to act as a receptor for these bacteria [7], placing the impetus for tetraspanin‐mediated adherence on their proximal proteins. *N. meningitidis* and *S. aureus* utilise different canonical receptors for adhesion suggesting that either differing CD9 platforms are required or that specific proteins are recruited to CD9 during adherence. To investigate this, we employed a proximity labelling methodology, fusing CD9 to the promiscuous biotin ligase, TurboID, which allows rapid labelling (1–10 min) and identification of proximal proteins (~10 nm) [23]. TurboID was fused to the C terminus of CD9 in line with previous strategies which have tagged CD9 [24]. A flexible linker was added to ensure access to the C terminus and allow free movement of TurboID (Fig. 2A). The construct also contains a V5‐tag at the TurboID C terminus as another method of detecting the protein. Stable transfection of A549 WT and CD9^−/−^ cells followed by western blotting revealed that the construct was expressed, with both endogenous CD9 and the CD9 fusion present in WT cells (Fig. 2B). Expression levels of the CD9:TurboID fusion protein were similar to those of the endogenous protein. Efficient biotinylation of a range of proteins at different molecular weights was observed after 10 min of addition of biotin to the media of transfected cells in comparison to WT and CD9^−/−^ cells (Fig. 2C). Continued accumulation of biotinylated proteins was observed between 1 and 18 h. Biotinylated proteins were efficiently pulled down using streptavidin beads from CD9^−/−^ transfected cells in comparison to CD9^−/−^ cells (Fig. 2D). Therefore, we have developed a model by which we can measure the proximal proteins of CD9 over time and with differing stimuli.

### CD9:TurboID fusion protein functions similar to endogenous CD9

**Fig. 3:**
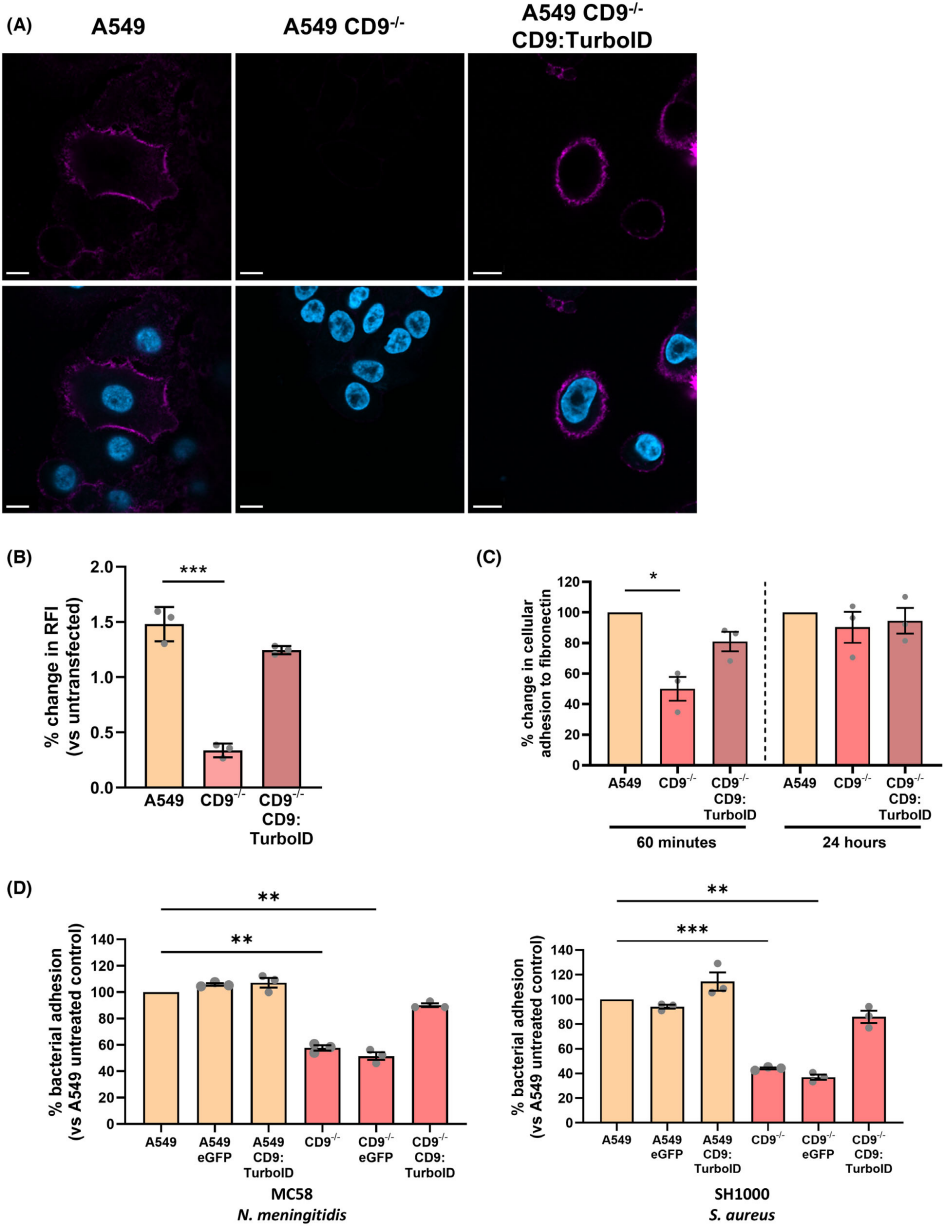
CD9:TurboID localises to the plasma membrane and rescues a meningococcal and staphylococcal infection phenotype. (A) Representative immunofluorescence images showing localisation of CD9 constructs. Wild‐type (WT) and CD9^−/−^ cells and those transfected with the CD9:TurboID fusion plasmid were fixed and treated with anti‐CD9 (602.29) antibodies. Localisation was visualised using a FITC‐conjugated secondary antibody. Scale bar signifies 10 μm. *n* = 3. (B) Cell surface expression of the CD9:TurboID fusion protein. WT, CD9^−/−^ and those transfected with the CD9:TurboID fusion plasmid were treated with an anti‐CD9 antibody (602.29). A mouse IgG isotype control (JC1) was included and expression was determined using a FITC‐conjugated secondary antibody. Relative fluorescence intensity was determined by dividing the median fluorescence intensity of the target protein by that of the isotype control. *n* = 3, mean ± SEM, one‐way ANOVA, ***P ≤ 0.001. (C) The cell adhesion phenotype of CD9 is rescued with expression of the CD9:TurboID fusion protein in CD9^−/−^ cells. Calcein AM labelled cells were allowed to adhere to fibronectin‐coated wells for 60 min or 24 h. *n* = 3, mean ± SEM, one‐way ANOVA, *P ≤ 0.05. (D) Meningococcal and staphylococcal infection phenotypes are rescued with expression of CD9:TurboID fusion protein in CD9^−/−^ cells. Cells were infected with meningococci (MC58) or staphylococci (SH1000) for 60 min at a multiplicity of infection (MOI) = 50. Cells were disrupted after infection and adherent, and internalised bacteria were enumerated by colony‐forming units (cfu). *n* = 3, mean ± SEM, one‐way ANOVA, **P ≤ 0.01, ***P ≤ 0.001.

To ensure that the CD9:TurboID fusion protein can function similarly to endogenous CD9, as alteration of the C terminus may affect normal function [25], we investigated both the localisation and biological activity of CD9 during bacterial infection. CD9 was abundantly expressed on the plasma membrane of WT cells in comparison to CD9^−/−^ or isotype control treated cells (Fig. 3A). Stably transfected CD9^−/−^ cells demonstrated a recovery of CD9 expression at the cell surface with both transfected cells and WT cells exhibiting strong expression at the plasma membrane with nonpunctate expression patterns. Recovered CD9 expression was confirmed in CD9^−/−^ cells transfected with the CD9:TurboID fusion plasmid by flow cytometry (Fig. 3B). CD9 expression was significantly reduced in CD9^−/−^ cells (0.34 ± 0.06) compared to WT cells (1.48 ± 0.16); however, CD9 expression was observed to be similar to that of WT cells (1.48 ± 0.16) in CD9^−/−^ cells transfected with the CD9:TurboID fusion plasmid (1.25 ± 0.04). Calcein AM assays were used to test the ability of cells to adhere to fibronectin over time. CD9^−/−^ cells demonstrated significantly reduced adherence to fibronectin over one hour (50.0 ± 13.4%) compared to WT cells; however, this phenotype was partially rescued through stable transfection with the CD9:TurboID fusion protein (80.9 ± 11.0%; Fig. 3C). No significant difference was observed in cell adhesion to fibronectin after 24 h. Epithelial cells were also checked for their ability to accommodate bacterial infection, as we have previously demonstrated that CD9 is important for multiple adherence pathways (Fig. 1). As expected, both meningococcal (57.7 ± 3.5%) and staphylococcal (44.1 ± 1.5%) adherence was significantly reduced in the CD9^−/−^ cells compared to WT and WT cells transfected with an eGFP construct (Fig. 3D). This was mirrored in CD9^−/−^ cells transfected with an eGFP construct. However, in CD9^−/−^ cells transfected with the CD9:TurboID construct, both meningococcal (90.0 ± 2.6%) and staphylococcal (85.6 ± 8.6%) adherence increased similar to levels observed in WT or eGFP transfected WT cells (Fig. 3D). Interestingly, despite overexpression of CD9 in CD9:TurboID transfected cells, only a small increase was observed in both meningococcal (107.1 ± 6.4%) or staphylococcal (114.4 ± 12.8%) adherence. We therefore demonstrate that CD9 can function normally despite fusion of TurboID to the C terminus.

### TurboID identifies several known CD9 interactors and significantly extends the number of putative proximal proteins

**Fig. 4:**
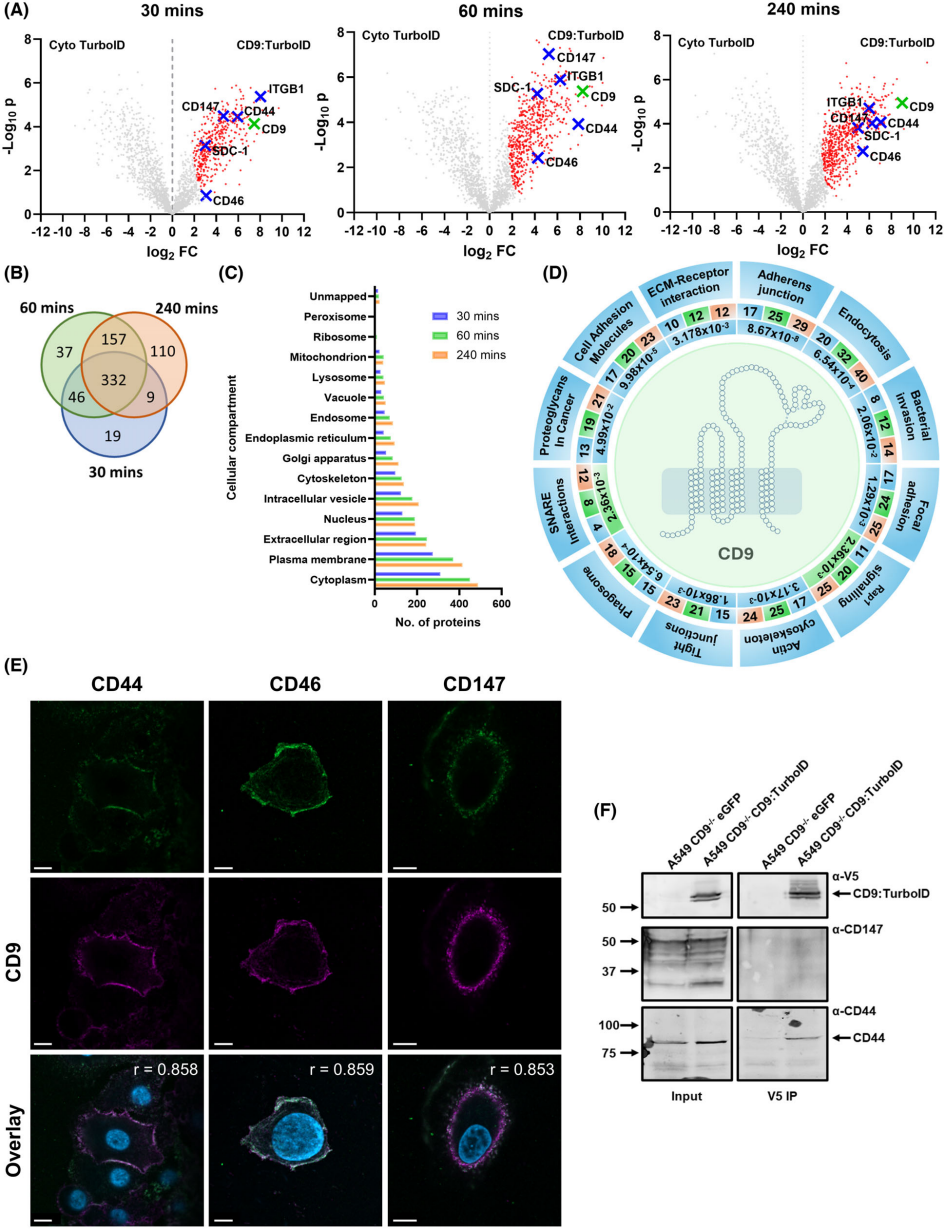
The CD9 interactome is large and encompasses several proteins involved in myriad cell functions. (A) Volcano plots of label‐free quantification (LFQ) data from CD9^−/−^ cells transfected with the CD9:TurboID fusion protein versus CD9^−/−^ cells transfected with a cytosolic TurboID treated with 50 μm biotin over time. Significantly enriched proteins (red) for the CD9:TurboID fusion protein were determined by Student's *t*‐test at a permutation‐based false discovery rate (FDR) of 0.01 and stringency (s0 = 2). Known meningococcal and staphylococcal receptors are shown (blue crosses) as is CD9 (green cross). *n* = 3. (B) Venn diagram demonstrating the overlap and dynamism of the CD9 interactome over time. Significantly enriched proteins were compared between timepoints, 30 min blue; 60 min green; 240 min orange. *n* = 3. (C) The majority of proximal proteins are found within the cytoplasm and the plasma membrane. Significantly enriched proteins over time were analysed through SubcellulaRVis and categorised by their cellular compartments. *n* = 3. (D) Selected biological functions associated with CD9 proximal proteins. Significantly enriched proteins were analysed by KEGG pathway. Selected enriched KEGG pathways (FDR ≤ 0.05) are shown. Numbers demonstrate proximal proteins associated with the pathway by time point, 30 min blue; 60 min green; 240 min orange. Inner ring shows FDR at the first time point demonstrating 95% confidence, colours as above. *n* = 3. (E) Representative images demonstrating colocalisation of CD9 with CD44, CD46 or CD147. Some images of CD9 expression in A549 cells from Fig. 3A have been reused here. Images show individual staining of CD9 or the target proteins and the overlay of the images with a DAPI nuclei stain. Pearson's co‐efficients are stated on overlay images. Scale bar signifies 10 μm. *n* = 3. (F) Whole cell lysates were immunoprecipitated with anti‐V5 antibodies; elutes were probed with anti‐CD44 or anti‐CD147 antibodies. Lysates from CD9^−/−^ cells transfected with eGFP were used as a control. *n* = 3.

To generate a definitive picture of the CD9 interactome, we purified biotinylated proteins over three time points, 30, 60 and 240 min, to capture proximal proteins involved with the proposed myriad functions of CD9. Time points were selected as critical times during early bacterial adherence and invasion and would allow later investigation of the dynamics of the CD9 interactome during bacterial infection. The volcano plots demonstrate the individual isolated proteins enriched through biotinylation by CD9:TurboID compared to a cytosol expressed TurboID control (Fig. 4A). 710 proteins were isolated across all three time points with 406, 572 and 608 proteins enriched at 30, 60 and 240 min, respectively (Fig. 4A,B). 166 enriched proteins were unique across the time points (30 min – 19; 60 min – 37; 240 min – 110), 212 were shared between two time points, and 332 proteins were shared across all three time points suggestive of a core interactome (Fig. 4B). The most enriched proteins were discoidin, CUB and LCCL domain containing protein 1 (*DCBCLD1*), an integral membrane protein, at 30 min, carboxypeptidase D (*CPD*), a type I membrane protein, at 60 min, and ephrin type‐A receptor 2 (*EPHA2*) at 240 min, a receptor tyrosine kinase anchored to the plasma membrane with roles in cell communication. Several known CD9 interactors were identified including CD9 itself, several integrins (α5 and β1), other tetraspanin members (CD151, Tspan15) and immunoglobulin superfamily members including IgSF3 (Fig. 4A, Data S1). Furthermore, several putative meningococcal and staphylococcal receptors were identified including CD147, CD46, CD44 and syndecan‐1 (Fig. 4A), which we have previously demonstrated directly interacts with CD9 [4]. CD46 is not within the enriched cohort of proximal proteins at 30 min, lying just outside of the stringency cutoff (s0 = 2), but is abundant at both 60 and 240 min. Despite changes in CEACAM expression after CD9 knockout (Fig. 1E), no CEACAMs were observed in the enriched proximal protein dataset.

Through analysis of the enriched CD9 interactome by cellular compartment, as expected most proteins observed were found in either the plasma membrane or the cytoplasm (240 min – 414 and 488 respectively) but a large number were also found in intracellular vesicles (240 min – 208; Fig. 4C). Many proteins were associated with the cytoskeleton (240 min – 138), which supports the role of CD9 in cell adhesion and migration but is also suggestive of our study isolating various signalling proteins associated with these processes. Interestingly, we also observed several proximal proteins associated with the extracellular region (240 min – 244; Fig. 4C). The number of proximal proteins associated with each cellular compartment rose over time except for those associated with the ribosome and peroxisome which both reduced at 240 min. Cellular localisation of the enriched proximal proteins demonstrates successful fusion and tagging of CD9.

Using a KEGG pathway analysis, we identified CD9 involvement in several cellular processes (Fig. 4D). Using a false discovery rate less than or equal to 0.05, 20 pathways were identified after 30 min, 28 after 60 min and 40 after 240 min (Data S2). Across all timepoints, several of these pathways have previously been linked with CD9 including adherens junctions, tight junctions, endocytosis and cell adhesion molecules [26] (Fig. 4D). Other expected pathways, such as SNARE interactions, were also observed (30 min, 4/33; 60 min, 8/33; 240 min, 12/33), with numbers of proteins involved with these pathways increasing over time. Despite no infectious challenge, proteins associated with several pathways involved in bacterial or viral infection were identified across all time points (30 min, 3; 60 min, 3; 240 min, 8), with specific correlations to *E. coli* invasion and *Yersinia* infection (Data S2). Numerous pathways often requisitioned by bacteria during infection were also identified including: (i) regulation of the actin cytoskeleton, critical in actin remodelling at the plasma membrane during infection; (ii) endocytic pathways; (iii) proteins associated with the formation of the phagosome; and (iv) ECM–receptor interaction, which includes important groups of proteins often commandeered for use during bacterial adherence. The latter contained several integrins and proteoglycans, previously identified as important during bacterial adherence, such as α5β1, CD44 and syndecan‐1 [4, 18, 22], but also other unidentified proteins such as dystroglycan‐1 which require further investigation. Interestingly, several signalling pathways were also identified during our proximity labelling screen; in particular, proteins associated with Rap1 signalling, a GTPase which modulates expression and activation of integrins and matrix metalloproteases [27], were observed from 60 min onwards (60 min, 20/210; 240 min, 25/210; Fig. 4D, Data S2). Therefore, using proximity labelling we have described the CD9 interactome, delineating several known proteins and novel CD9 interactors while simultaneously identifying a role for CD9 in both canonical and noncanonical pathways.

To confirm the interactions identified in this screen, colocalisation and coimmunoprecipitation assays were carried out targeting the putative bacterial receptors identified and CD9. High levels of colocalisation between CD9 and CD44 (*r* = 0.858), CD46 (*r* = 0.859) and CD147 (*r* = 0.853) were observed at the plasma membrane (Fig. 4E). Staining of all four proteins was observed uniformly across the plasma membrane, however, there appear to be clear areas of colocalisation for each of these proteins. Co‐immunoprecipitation of CD44 with the overexpressed CD9:TurboID fusion protein confirmed this data (Fig. 4F). An interaction of CD9 with CD147 could not be validated by co‐immunoprecipitation suggesting either a transient or weak interaction between these proteins. Further experiments are required to check milder detergents as interactions may occur through additional partner proteins. CD46 has previously been demonstrated to associate with CD9 and β1 integrins [21], and therefore, our studies focused on co‐immunoprecipitation of CD44 and CD147 with CD9. These data demonstrate interactions of these proteins through both colocalisation and coimmunoprecipitation studies, however, these interactions may be transient or indirect in regards to CD147.

### The CD9 interactome is dynamic and changes with bacterial infection

**Fig. 5:**
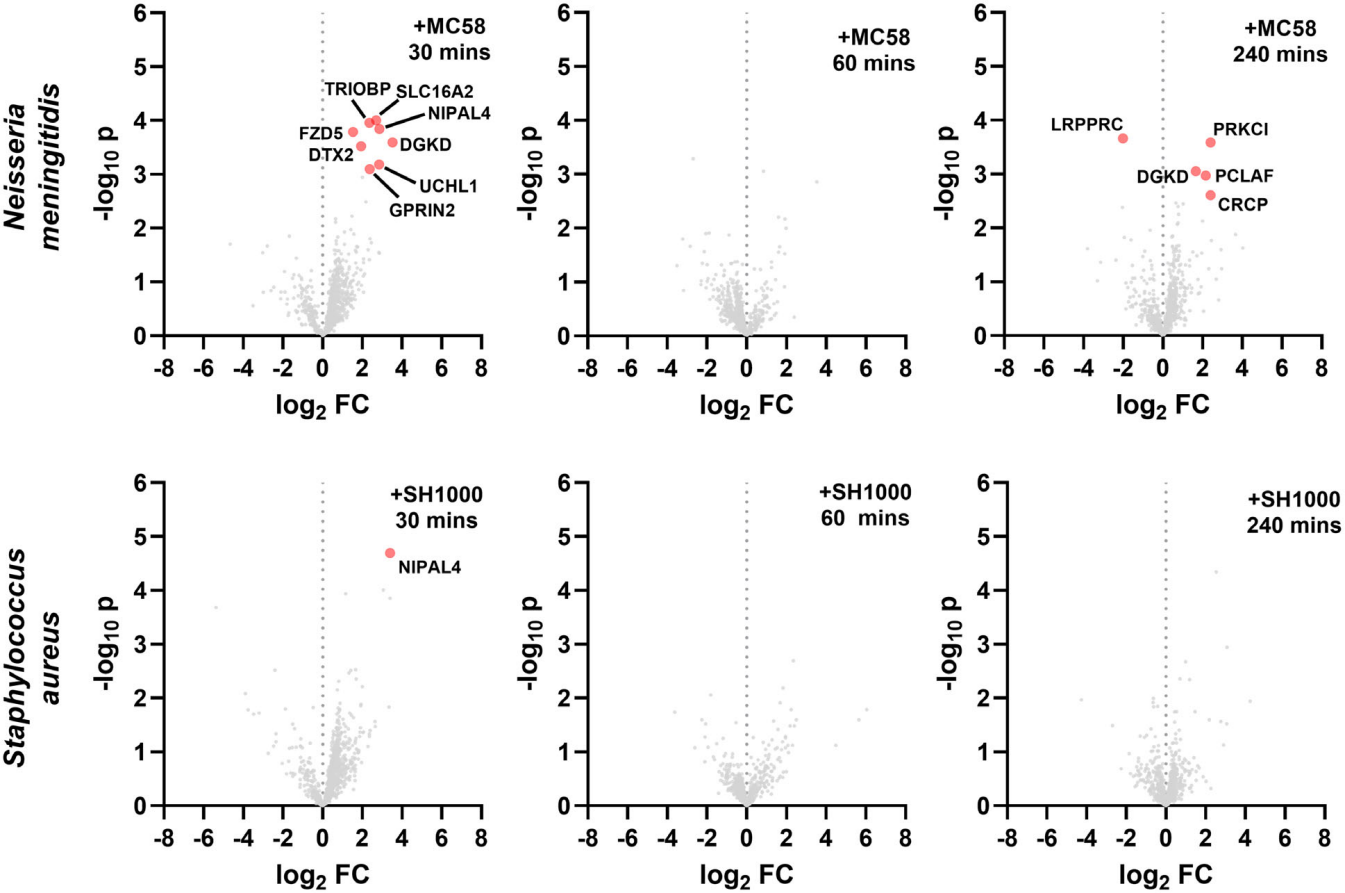
The CD9 interactome is dynamic and changes dependent on infection. Volcano plots of label‐free quantification (LFQ) data from infected CD9^−/−^ cells transfected with the CD9:TurboID fusion protein versus uninfected CD9^−/−^ cells transfected with the CD9:TurboID fusion protein. Cells were treated with 50 μm biotin and infected with either meningococci (MC58) or staphylococci (SH1000) at an MOI = 50 for the stated time period. Data sets were initially compared to proteins enriched from CD9^−/−^ cells transfected with a cytosolic TurboID construct to remove non‐specific TurboID labelled proteins. Initial filtering determined enriched proteins by Student's *t*‐test with a false discovery rate (FDR) of 0.01 and s0 = 2. Filtered datasets were compared and significantly enriched proteins (red) were determined by Student's *t*‐test with a permutation‐based FDR of 0.05 and of stringency (s0 = 0.1). *n* = 3.

As we have observed that numerous proteins associated with bacterial adherence pathways are enriched during our CD9 proximity labelling screen and that interference with CD9 can diminish bacterial adherence, we have investigated whether the CD9 interactome can change during meningococcal or staphylococcal adherence. The volcano plots demonstrate that twelve proteins proximal to CD9 are enriched during meningococcal infection in comparison to uninfected cells (30 min, 8; 60 min, 0; 240 min, 4; Fig. 5). The most enriched proteins associated with meningococcal infection were Solute carrier family 16 member 2 (*SLC16A2*) at 30 min and protein kinase C iota type (*PRKCI*) at 240 min. One protein, diacylglycerol kinase delta (*DGKD*), was enriched at both 30 and 240 min. Conversely, one CD9 proximal protein, LRP 130 (*LRPPRC*), was enriched in uninfected cells compared to cells infected with meningococcal bacteria at 240 min. In contrast, only one protein that associates with CD9 was enriched during staphylococcal infection after 30 min, magnesium transporter NIPA4 (*NIPAL4*; Fig. 5). Magnesium transporter NIPA4 was also enriched during meningococcal infection at 30 min but all other proteins identified were unique to the specific infecting bacteria. Specific functions for the identified proteins are provided (Table 1).

**Table 1:** Functions of significantly enriched CD9 proximal proteins observed during infection. Proteins enriched in meningococcal‐infected cells (orange), or cells infected with either meningococci or staphylococci (green) vs. proteins enriched in uninfected cells (blue).

Gene	Full name	Function	Classification	
*DGKD*	Diacylglycerol kinase delta	Phosphorylates diacylglycerol to produce phosphatidic acid	PI signalling pathway	30 min
*DTX2*	Deltex E3 ubiquitin ligase 2	E3 ubiquitin ligase	Notch signalling pathway	
*FZD5*	Frizzled class receptor 5	G protein coupled receptor, involved in Wnt signalling	Wnt signalling pathway	
*GPRIN2*	G protein regulated inducer of neurite outgrowth 2	Involved in neurite outgrowth, active at the plasma membrane	Protein binding	
*NIPAL4*	NIPA like domain containing 4	Mg^2+^ transporter	Ion transport	
*SLC16A2*	Solute carrier family 16 member 2	Thyroid hormone transmembrane transporter	Transport	
*TRIOBP*	TRIO and F‐actin binding protein	Interacts with TRIO, stabilises F‐actin	Cytoskeletal proteins	
*UCHL1*	Ubiquitin C‐terminal hydrolase L1	Deubiquitinase involved in regulation of inflammation	Ubl conjugation pathway	
*CRCP*	CGRP receptor component	Calcitonin receptor increases cAMP levels	Transcription machinery	240 min
*DGKD*	Diacylglycerol kinase delta	Phosphorylates diacylglycerol to produce phosphatidic acid	PI signalling pathway	
*PCLAF*	PCNA clamp‐associated factor	Regulator of DNA repair	DNA repair	
*PRKCI*	Protein kinase C iota type	Serine/threonine protein kinase, protective role in apoptosis	Serine/threonine protein kinase	
*LRPPRC*	Leucine rich pentatricopeptide repeat containing	Binds and traffics poly(A) mRNA	Transcription regulation	

Several of these proximal proteins are involved in typically appropriated pathways during infection, including cytoskeletal rearrangement, membrane trafficking, transport and signalling. 9/12 proteins were uniquely enriched at their specific timepoint; however, TRIO and F‐actin‐binding protein (*TRIOBP*) and magnesium transporter NIPA4 were enriched in infected cells at 30 min, but were also observed in untreated cells at 60 and 240 min (*TRIOBP*) or just at 240 min (NIPA4; Data S1), suggesting bacterial infection induces recruitment to sites proximal to CD9 earlier than usual. TRIO and F‐actin‐binding protein is involved in cytoskeletal reorganisation and protein trafficking [28], while magnesium transporter NIPA4 is a membrane‐associated Mg^2+^ transporter associated with skin barrier function and epidermal lipid processing [29] suggesting an earlier recruitment could facilitate meningococcal and staphylococcal induced membrane perturbation. Further membrane‐associated proteins have been enriched after 30 min of meningococcal infection including Solute carrier family 16 member 2, a solute transporter, and GRIN2 (*GPRIN2*) which has shown involvement in MAPK and cytokine signalling and membrane projection [30]. The G protein coupled receptor, Frizzled‐5 (*FZD5*), the deubiquitinase, Ubiquitin carboxy‐terminal hydrolase isozyme L5 (*UCLH1*), and diacylglycerol kinase delta are all associated with inflammatory signalling pathways [31, 32] suggesting interaction of CD9 with proteins involved in regulating the inflammatory response during early meningococcal infection of epithelial cells. Interestingly, E3 ubiquitin‐protein ligase (*DTX2*), a protein involved in DNA damage responses, was also enriched after 30 min of meningococcal infection.

At 240 min post‐meningococcal infection, proteins involved in DNA damage responses (PCNA‐associated factor (*PCLAF*)) and inflammation and immune cell activation (CGRP receptor component protein (*CRCP*), diacylglycerol kinase delta and protein kinase C iota type) were enriched compared to uninfected cells. Diacylglycerol kinase delta was enriched at both 30 min and 240 min post‐meningococcal infection suggesting an important role for this protein throughout CD9‐mediated infection. CGRP receptor component protein, a protein involved in attenuating innate immune responses during bacterial infection [33], was also enriched at 240 min and was just below the stringency threshold at 30 min further suggesting CD9 involvement in dampening the meningococcal inflammatory response. Finally, we observed a reduction in the enrichment of LRP 130, a protein which has shown extensive involvement in the regulation of inflammatory responses during viral infections [34]. Overall, we demonstrate that the CD9 interactome can change during bacterial infection, but these changes are dependent on the infectious agent.

### The CD9 interactome includes known meningococcal and staphylococcal receptors which are involved in CD9‐mediated bacterial adherence

**Fig. 6:**
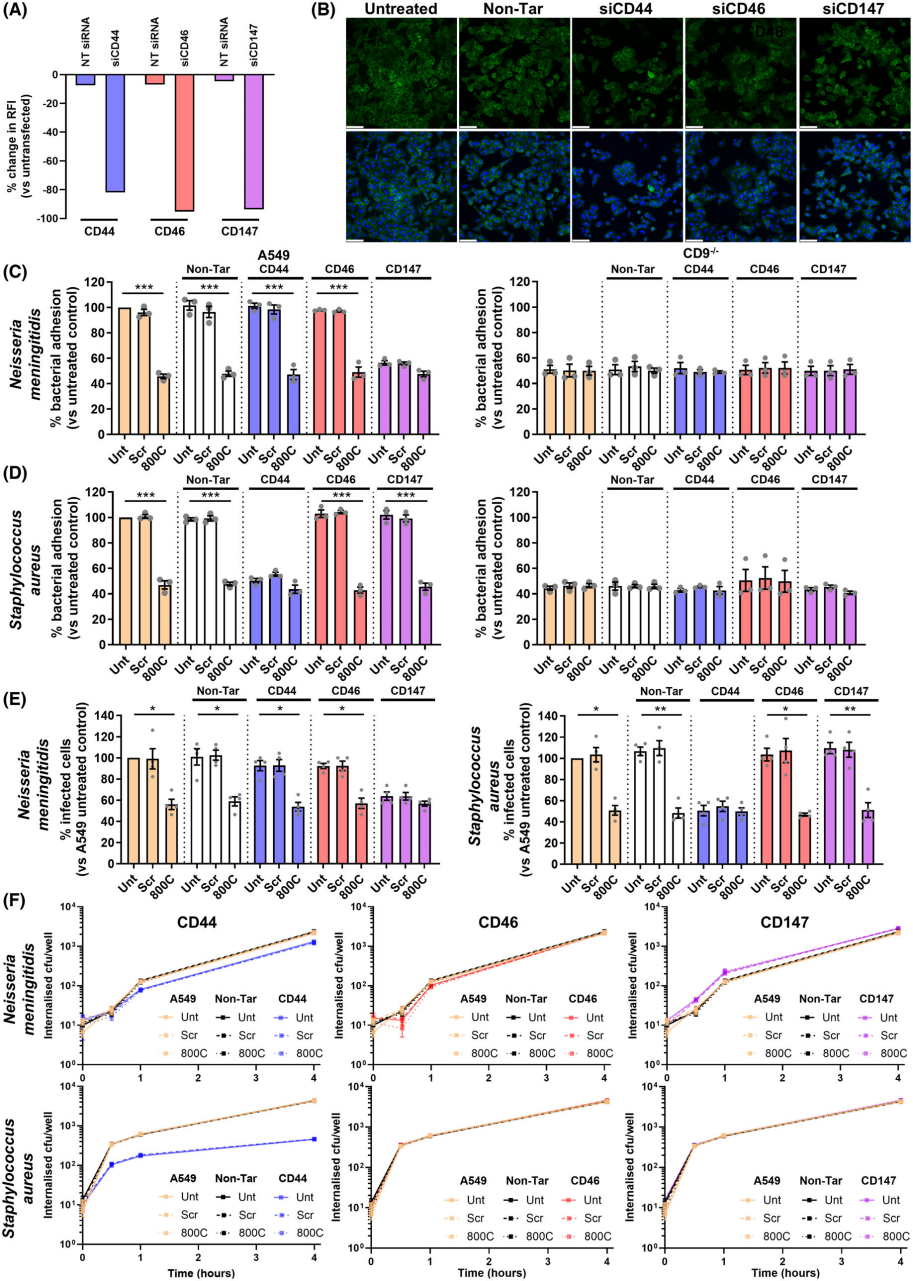
Differing CD9 proximal proteins are involved in CD9‐mediated meningococcal and staphylococcal adhesion. Wild‐type (WT) cells were treated with specific siRNAs 72 h prior to infection. (A) Flow cytometry demonstrating efficient knockdown of meningococcal and staphylococcal receptors after siRNA treatment prior to infection compared to cells treated with nontargeting siRNA (NT). Cells were probed with anti‐CD147, anti‐CD46 and anti‐CD44 antibodies. *n* = 1, mean. (B) Representative microscopy images demonstrating no effect on CD9 expression or localisation by siRNA‐mediated knockdown of CD44, CD46 or CD147. Scale bars signify 100 μm. *n* = 3. (C) CD147 knockdown and CD9‐derived peptide treatment demonstrate no additive effects on meningococcal adherence. Cells were treated with 800C or a scrambled control peptide (Scr) for 60 min prior to infection. WT and CD9^−/−^ cells were infected with meningococci (MC58) for 60 min at an MOI = 50. Untreated cells denoted as Unt, nontargeting siRNA treatment designated as Non‐Tar. *n* = 3, mean ± SEM, one‐way ANOVA, ***P ≤ 0.001. (D) CD44 knockdown and CD9‐derived peptide treatment demonstrate reduced additive effects in staphylococcal adherence. WT and CD9^−/−^ siRNA‐treated cells were infected with staphylococci (SH1000) for 60 min at MOI = 50. Cells were disrupted after infection, and adherent and internalised bacteria were enumerated by colony‐forming units (cfu). *n* = 3, mean ± SEM, one‐way ANOVA, ***P ≤ 0.001. (E) CD147 and CD44 demonstrate involvement in CD9‐mediated meningococcal and staphylococcal adherence, respectively, by microscopy. WT siRNA‐treated cells were infected with meningococci (MC58) and staphylococci (SH1000) for 60 min at MOI = 50. Cells were stained with Giemsa, and adherent bacteria were enumerated by light microscopy. *n* = 4, mean ± SEM, one‐way ANOVA, *P ≤ 0.05, **P ≤ 0.01. (F) Knockdown of bacterial receptors affects bacterial internalisation but CD9‐derived peptides have no effect. WT siRNA‐treated cells were infected with meningococci or staphylococci for 60 min at MOI = 50 at 4 °C for 60 min. Cells were washed and internalisation was allowed to proceed for 4 h at 37 °C. External bacteria were killed with gentamicin or lysostaphin for one hour before cells were washed and lysed. Internalised bacteria were enumerated by cfu counts. *n* = 3 mean ± SEM, Friedman test.

We specifically demonstrate enrichment of several known meningococcal and staphylococcal receptors amongst the CD9 interactome. As we and others have previously demonstrated direct CD9 interaction of β1 integrins and syndecan‐1 in staphylococcal adhesion [4, 35], two important hits from our screen, we wanted to further demonstrate that CD9 and other receptor candidates from the interactome were involved in tetraspanin‐mediated bacterial adherence and that this was dependent on the infecting bacteria. We therefore focused on CD46 and CD147 as putative pilus receptors of the meningococcus and CD44 as a known interactor in various bacterial adherence pathways including *Escherichia coli* [36], group A Streptococcus (GAS) [37] and *S. aureus* [22]. Successful knockdown of each of our target proteins using pooled siRNA was demonstrated (Fig. 6A). Transient knockdown of the target proteins had no effect on CD9 expression or localisation within cells (Fig. 6B). After siRNA‐mediated knockdown of these proteins, we observed significant reductions in meningococcal adherence to CD147‐depleted epithelial cells (56.4 ± 3.1%) compared to untreated or nontargeting siRNA‐treated cells (Fig. 6C). CD46 or CD44 knockdown did not impact meningococcal adherence to epithelial cells suggesting the importance of CD147, but not CD46 or CD44. As observed previously, addition of a CD9‐derived peptide (800C) to untreated and nontargeting siRNA‐treated cells significantly reduced meningococcal adherence (45.7 ± 3.0% and 47.8 ± 3.6%, respectively) but no effect was demonstrated after treatment with a scrambled control peptide (Fig. 6C). This significant reduction was also observed in CD46‐ and CD44‐depleted cells. However, no significant reduction was observed between CD147‐depleted cells with no peptide treatment and CD147‐depleted cells treated with 800C. This suggests no major additive effect of these treatments and that CD147 and CD9 interact within the same pathway during meningococcal adherence. As before, meningococcal adherence to CD9^−/−^ cells was significantly reduced compared to the WT cells (51.1 ± 5.3%; Fig. 6C). However, as with all siRNA treatments and peptide treatments, CD147 depletion had no effect on meningococcal adherence in CD9^−/−^ cells (Fig. 6C), further suggesting the importance of interaction between these two proteins during tetraspanin‐mediated adherence.

Similarly, significant reductions were observed in staphylococcal adherence to CD44‐depleted cells (50.4 ± 3.0%) compared with untreated and nontargeting siRNA‐treated cells (Fig. 6D). However, no effects were demonstrated in CD46‐ or CD147‐depleted epithelial cells suggesting CD44 is important during staphylococcal adherence. Upon addition of 800C, staphylococcal adherence was significantly reduced in untreated cells (47.0 ± 5.6%), nontargeting siRNA cells (47.8 ± 2.4%), CD46‐ (42.9 ± 4.2%) and CD147‐depleted cells (45.6 ± 4.9%; Fig. 6D). Addition of a scrambled control peptide had no effect on any of the siRNA‐treated cells. However, no significant reduction in adherence was observed when CD44‐depleted cells with no peptide were compared to CD44‐depleted cells treated with 800C. As before, this suggests that the interaction between CD44 and CD9 is important during staphylococcal adherence. Staphylococcal adherence was significantly reduced in CD9^−/−^ cells compared to wild‐type cells but no further reductions were observed with any siRNA or peptide treatments, including CD44‐depletion (Fig. 6D). These data further suggest the importance of an interaction between CD9 and CD44 during tetraspanin‐mediated staphylococcal adherence. Functional interactions between CD147 and CD9 in meningococcal adherence and CD44 and CD9 during staphylococcal adherence were confirmed through adherent bacteria counts using light microscopy (Fig. 6E).

We further investigated the effects of siRNA‐mediated knockdown in combination with CD9 blockade on bacterial internalisation over four hours. CD44 knockdown demonstrated a small nonsignificant reduction in meningococcal internalisation (4 h – 1313.3 ± 228.1 cfu) in comparison to the untreated WT epithelial cells (4 h – 2113.3 ± 228.1 cfu; Fig. 6F). Conversely, a small nonsignificant increase in meningococcal internalisation was observed in CD147‐depleted cells (4 h – 2796.7 ± 176.1 cfu) in comparison to the untreated WT cells. No change was observed in CD46‐depleted cells or those treated with nontargeting siRNA. Furthermore, no effect was observed on meningococcal internalisation when cells were treated with scrambled or 800C peptide (Fig. 6F). Similarly, CD46‐ and CD147‐depleted cells demonstrated no significant changes to staphylococcal internalisation over time similar to cells treated with nontargeting siRNA (Fig. 6F). Again, singular treatments of scrambled or 800C peptide or those in combination with depleted cells had no effect on staphylococcal internalisation. Depletion of CD44 demonstrated a large but nonsignificant reduction (4 h ‐ 455 ± 47.7 cfu; *P* = 0.6290) in staphylococcal internalisation in comparison to untreated WT cells (4 h ‐ 4426.7 ± 327.2 cfu; Fig. 6F). This suggests that while individual receptors may have effects on internalisation, CD9 organisation of these proteins is not necessary downstream of the adhesion process. In conclusion, we have demonstrated that known meningococcal and staphylococcal receptors, identified within our CD9 proximal protein screen, are important during tetraspanin‐mediated bacterial adherence and that, critically, these proximal proteins differ between the infecting organism but CD9 remains as a universal constant during bacterial adherence.

## Discussion

In this study, we have characterised the CD9 interactome using a proximity labelling approach with the promiscuous biotin ligase, TurboID, fused to CD9. We demonstrate that the fusion of TurboID to the C terminus of CD9 has no effect on CD9 localisation and function, and the use of the fusion protein reestablishes bacterial adherence to cells lacking CD9. We have identified several novel and known enriched proximal proteins associated with CD9. Many of these proximal proteins are involved in mammalian cell adhesion and their associated signalling pathways; however, there is also a prevalence for proteins utilised during bacterial adherence and invasion pathways. We demonstrate enrichment and recruitment of specific proteins during meningococcal adherence but not during staphylococcal adherence, suggesting dynamic and mobile CD9 microdomains during tetraspanin‐mediated bacterial adherence. Finally, we demonstrate the involvement of CD147 and CD44 in CD9‐mediated meningococcal and staphylococcal adherence respectively, but not vice versa confirming the requirement for specificity of proximal proteins during tetraspanin‐mediated bacterial adherence.

Several studies have demonstrated that the tetraspanins interact with myriad partner proteins [38]; however, these studies have often focused on the interaction of the tetraspanin with a specific partner. Various techniques have been used to populate a partial interactome for CD9 and the other tetraspanins, including immunoprecipitation [4], super resolution microscopy [39, 40], single molecule tracking [41], cryo‐EM [42], yeast two‐hybrid assays [43] and affinity purification [44]. However, these techniques often require knowledge of potential tetraspanin interactors, require high levels of expression of the target proteins, struggle to evaluate weak or transient interactions and provide limitations to the screen size. Recently, attempts have been made to broadly define the tetraspanin interactome using proximity labelling techniques [45]. Proximity labelling using TurboID followed by mass spectrometry allows rapid biotinylation of proximal proteins to the target protein, providing a broad interactome over time and after specific challenges to the cell which also considers weak or transient interactions [23]. We have developed a tool to measure the CD9 interactome in epithelial cells by fusing TurboID to the C terminus of CD9.

We believe this is the first study of the broader CD9 interactome by proximity labelling, identifying 710 potential proximal proteins after incubation with exogenous biotin for 4 h. This included known interactors such as IGSF3 [46], HSPGs [4], various integrins [19], metalloproteases [47] and other tetraspanins but also several novel interactors involved in signal transduction, transportation and cell migration. Similarly, Cheerathodi *et al*. identified 1600 proteins within the CD63 interactome after 36 h with enrichment of Rab GTPases and SNARE proteins [45]. While we observed similar pathways and proteins, we also describe enrichment of many tight and adherens junction proteins, perhaps unsurprising considering the canonical localisation of CD9 to the plasma membrane in comparison to CD63 localisation within the lysosome. Surprisingly, we detected enrichment of several extracellular proteins; however, we suspect that a cohort of these proteins is not included within our interactome due to fusion of TurboID to the cytoplasmic tail of CD9. For example, despite the robust presence of cell membrane surface HSPGs, such as syndecan‐1 and CD44, within our screen we did not detect any glycosylphosphatidylinositol (GPI)‐anchored proteins, such as the glypicans or specific members of the CEACAM family. However, the lack of enrichment of any CEACAM members, either GPI‐anchored or otherwise, suggests that CD9 does not directly associate with these proteins but they may be indirectly associated with the tetraspanin through more transient interactions within the cell membrane. Future studies could focus on CD9‐interacting extracellular proteins by utilising new proximity labelling techniques [48]. However, CD9 enrichment of both membrane proteins and effectors associated with cell signalling cascades provides further evidence of the role of CD9 as a coordinator of the plasma membrane but also downstream proteins and cellular processes.

We have previously demonstrated that CD9 does not act as a bacterial receptor and that their partner proteins are required for efficient bacterial adherence [4, 7], similar to viral utilisation of the tetraspanins [49]. Here, we confirmed, through transient knockdown, co‐immunoprecipitation and colocalisation, that two highly enriched CD9 proximal proteins, CD147 and CD44, were associated with CD9‐mediated meningococcal and staphylococcal adherence, respectively. CD147 is a known meningococcal receptor [14] while CD44 has been demonstrated as a phagocytic receptor of *S. aureus* in macrophages [22] and was recently enriched during a *S. aureus* proximity labelling screen of endothelial cell infection [50]. This, alongside our previous data that two other significantly enriched proximal proteins, α5β1 integrin and syndecan‐1, are involved in CD9‐mediated staphylococcal adherence [4], suggests that proximity labelling of CD9 proximal proteins enriches numerous candidate proteins during bacterial infection. However, we observed no change in meningococcal adherence after transient knockdown of CD46 suggesting no involvement of this putative receptor [15] in CD9‐mediated bacterial adherence despite enrichment of the protein at both 60 and 240 min in uninfected cells.

As previously observed by ourselves and others [4, 7, 8, 51], interference of CD9 or transient knockdown of CD147 and CD44 reduced bacterial association to epithelial cells by approximately 50%. As bacteria have a number of mechanisms to adhere to and enter cells, we believe it is likely that there are several mechanisms of bacterial adherence that CD9 is not involved in which would allow a number of bacteria to still adhere to cells. For example, we have not observed an interaction of CD9 with CEACAMs which are major receptors for meningococci and other bacteria [16]. There is also a level of redundancy between the other tetraspanins which has been reported in studies using antibodies to interfere with the tetraspanins and bacterial adherence [7, 52]. However, our recent studies have demonstrated that the CD9‐derived peptides are specific to CD9 and that the use of similarly derived peptides for CD81 had no effect on adherence [4]. It has previously been reported that knockdown of CD147 can reduce adherence to endothelial cells by approximately 80% [53]. We do not observe this level of efficacy; however, our study uses a different cell type, and we double the infection time described within the previous study.

Anti‐adhesion therapies for bacteria could potentially reduce the bacterial burden of disease in more complex models allowing for clearance of bacteria by immune responses before the initiation of infection [54]. To address whether CD9 has further effects downstream of bacterial adherence to epithelial cells, we measured the effects of CD9 interference and knockdown of CD44, CD46 and CD147 on internalisation. However, while CD44 knockdown demonstrated a reduction in staphylococcal invasion and small changes in meningococcal internalisation were associated with CD147 and CD46 knockdown, no effect was observed with CD9 interference. This suggests that CD9 is not involved in the invasion processes beyond the initial adherence to the epithelial cell of *S. aureus* and *N. meningitidis*. Interestingly, CD9 has no effect on the adhesion of canonical intracellular pathogens like *Burkholderia* spp. [10, 11] but does affect internalisation. This dichotomy suggests differing adherence and internalisation pathways for bacteria with interchangeable roles for CD9 dependent on the bacterial characteristics.

Similar to viral remodelling of TEMs [45], we observed dynamism in CD9‐enriched microdomains; however, enrichment of cognate partner proteins was aligned with infection by specific bacteria. At both 30 min and 240 min, infection with meningococci led to more enriched CD9 proximal receptors and downstream effectors than infection with staphylococci. We hypothesise that the required receptors for staphylococcal infection, particularly those required for bacterial adhesion after 30 min, are likely within preformed CD9‐enriched microdomains, and thus, its recruitment is less dynamic than during meningococcal infection. For example, we observed high enrichment of both α5β1 integrin and syndecan‐1 within uninfected cells, which are linked to canonical and noncanonical staphylococcal adherence pathways [4, 18]. Interestingly, Magnesium transporter NIPA4 was the only protein enriched during both meningococcal and staphylococcal infection, although, we note that PCNA‐associated factor was also present during staphylococcal infection but just outside the significance threshold. Magnesium transporter NIPA4 has not been associated with the tetraspanins previously, and no studies have suggested a role for this protein during infection; however, these data suggest recruitment of this transporter by two distinct bacteria at early time points which merits further investigation.

Our study also demonstrated enriched proteins involved in pathways utilised by both bacteria and viruses to enter cells and provided similar partner proteins to those previously observed in other tetraspanin studies. For example, during HCV entry to the cell, the virus binds directly to CD81 and triggers epidermal growth factor receptor (EGFR) signalling pathways critical for endocytosis of the virus [55]. Interestingly, during meningococcal adherence, we observed enrichment of diacylglycerol kinase delta at both 30 and 240 min, which regulates EGFR through modulation of PKC signalling [56], a member of which was also enriched at 240 min (protein kinase C iota type). The enrichment of CD9 proximal proteins involved in cell signalling and cytoskeletal regulation in this study would suggest significant remodelling of the architecture and organisation of the plasma membrane during tetraspanin‐mediated infection. The CD9 interactome contained several tight and adherens junction proteins. Bacterial‐induced remodelling of the cell surface, particularly of basolateral proteins involved in tight junctions, can promote bacterial traversal, particularly of the blood–brain barrier [57]. We observed no changes in internalisation of bacteria after treatment of cells with CD9‐derived peptides suggesting that instead of manipulating intracellular traversal routes, bacteria could utilise CD9 to disrupt tight junctions and manipulate paracellular traversal routes. We therefore suggest multiple mechanisms by which bacteria could manipulate tetraspanins for adherence and potentially invasion and may explain their involvement in many bacterial infections [7, 9, 11].

Meningococci possess a robust phase variation mechanism allowing rapid switching of expression states for many virulence factors critical for bacterial adherence allowing association with various host surface proteins [58]. Therefore, in the absence of a known effector molecule released by the bacteria to signal for membrane composition changes, and the speed of bacterial adherence within our model, changes in bacterial outer membrane virulence factors could cause shifts within CD9‐enriched microdomains over time. Future studies could utilise proximity labelling techniques which provide better temporal resolution of rapid changes, such as the use of APEX or APEXII [59]. This may delineate further dynamics within CD9‐enriched microdomains as biotinylation begins upon the addition of hydrogen peroxide therefore allowing specific timepoints and protein localisation to be addressed, however, the addition of hydrogen peroxide may have a detrimental effect on the bacteria.

We have successfully generated a tool to track the CD9 interactome in epithelial cells using proximity labelling and demonstrated dynamism within these microdomains in response to infection with either *N. meningitidis* or *S. aureus*. We demonstrate a robust CD9 interactome with proximal proteins involved in numerous cellular pathways. Furthermore, CD9‐mediated adherence required host cell surface receptors already present within the interactome but also recruitment of various proteins dependent on the infecting bacteria. Therefore, we present for the first time: (i) a replete CD9 interactome within epithelial cells; (ii) demonstrate the utility of CD9 as a universal organiser of ‘adhesion platforms’ for bacteria; (iii) and provide new targets for investigation within bacterial adherence pathways and their downstream signalling responses.

## Materials and methods

### Strains and bacterial growth conditions

The *N. meningitidis* and *S. aureus* strains used in this study were serogroup B MC58 (a kind gift from Robert Read, University of Southampton, [60]) and SH1000 (a kind gift from Peter Monk, University of Sheffield, [61]) respectively. *N. meningitidis* solid cultures were grown on brain:heart infusion (BHI; Oxoid, Ltd., Basingstoke, UK) agar with 10% Levinthal's solution [62] overnight at 37 °C with 5% CO_2_, while staphylococcal strains were grown on Luria broth (LB; Oxoid) agar overnight at 37 °C. Meningococcal liquid cultures were grown in BHI broth with 5% Levinthal's solution and 10 mm sodium bicarbonate at 37 °C with constant agitation. SH1000 was grown in LB at 37 °C with constant agitation. All liquid cultures were inoculated using freshly grown plates.

### Cell culture

Wild‐type (WT) and CD9 knockout (CD9^−/−^) A549 human lung epithelial cells (a kind gift from David Blake (Fort Lewis College, Colorado, USA) [63]) were maintained in Dulbecco's modified Eagle's media (DMEM; Thermo Fisher Scientific, Waltham, MA, USA) and 10% heat‐inactivated fetal calf serum (FCS; Gibco, Waltham MA, USA). All experiments were performed with mycoplasma‐free cells.

### Peptides and antibodies

The CD9 EC2‐derived peptide (800C; DEPQRETLKAIHYALN) and scrambled peptide (Scr; QEALKYNRAETPLDIH) were designed as described previously [4] and synthesised using solid phase Fmoc chemistry (GenScript Inc., Piscataway, NJ, USA). The peptide, 800C, has been demonstrated to inhibit staphylococcal interactions with human cells without affecting bacterial growth [4].

Mouse anti‐human CD9 IgG1 (MM2/57; Merck, Darmstadt, Germany), mouse anti‐human CD9 IgG1 (602.29; kind gift of Lynda Partridge, University of Sheffield, Sheffield, UK), mouse anti‐human CD147 IgG1 (HIM6; Biolegend, San Diego, CA, USA), mouse anti‐human CD46 IgG1 (MEM‐258; Thermo Fisher Scientific), mouse anti‐human CEACAM1 IgG1 (B3‐17; Merck), mouse anti‐human CEACAM6 IgG1 (1H7‐4B; Merck), mouse anti‐human CD44 IgG2a (60224‐1‐Ig; Proteintech Group, Inc, Rosemont, IL, USA), mouse anti‐human GAPDH (MAB374; Merck), mouse IgG1 (JC1; in house), mouse IgG2a (02‐6200; Thermo Fisher Scientific), mouse anti‐FLAG (M2; Merck), goat anti‐mouse HRP (P0447; Agilent, Santa Clara, CA, USA) were used as described.

### Flow cytometry

Flow cytometry was used to measure tetraspanin and bacterial receptor expression. Cell dissociation buffer (Thermo Fisher Scientific) was used to detach adherent cells before labelling with the relevant antibody at 4 °C for 60 min. If required, cells were secondary labelled with a fluorescein isothiocyanate (FITC) conjugated goat anti‐mouse IgG antibody (F5897, Merck). Cells were fixed with 1% paraformaldehyde, quantified with an LSRII cytometer (Becton Dickinson, Oxford, UK) and analysed by flowjo v10.0.7r2 software (BD).

### Western blots

Cell lysates were prepared in RIPA buffer (10 mm Tris/HCl pH8.0, 140 mm NaCl, 0.5 mm EGTA, 1 mm EDTA, 0.1% sodium deoxycholate, 0.1% SDS, 1% Triton X‐100) with a protease inhibitor cocktail (Complete Mini, Roche Ltd., Basel, Switzerland) and fractionated on SDS‐PAGE gels. Fractionated proteins were blotted on to a nitrocellulose membrane, blocked with nonfat‐dried milk diluted in TBST and subsequently probed with the appropriate antibody and visualised using an ECL detection system (Merck). To blot for biotinylated protein, nitrocellulose blots were blocked in 5% BSA and probed with a streptavidin HRP conjugate (Thermo Fisher Scientific).

### Plasmids

The lentiviral vector used to express CD9‐TurboID in our study was constructed and packaged by Vectorbuilder (VB220615‐1144dtm; Vectorbuilder Inc., Chicago, IL, USA). The vector was designed to fuse TurboID to human CD9 at the C terminus separated by a 3 tandem GGGGS linker under the control of a CMV promoter. A V5 tag was added to the C terminus of TurboID. An eGFP lentiviral control vector (VB010000‐9389rbj; Vectorbuilder) was used throughout the study. Addition of the puromycin resistance gene allowed for selection and maintenance of cells containing the construct. Lentiviral transfection was carried out as previously described [4]. Recombinant lentivirus was produced by co‐transfecting subconfluent HEK293T cells with the pMD2G (Addgene, Watertown, MA, USA), psPAX2 (Addgene) and pLV[Exp]‐CD9‐TurboID or the pLV[Exp]‐eGFP vectors with jetPEI (Polyplus transfection, Illkirch‐Graffenstaden, France). Media was changed after 24 h, and lentivirus‐containing supernatants were harvested after 48 h. For stable transfection, WT or CD9^−/−^ cells were grown for 24 h, 20% of culture media was replaced with lentivirus‐containing supernatant. Polybrene was used to increase transduction efficiency. Transduction was checked by protein electrophoresis and western blotting.

Nonspecific cytosolic TurboID labelling was assessed with V5‐TurboID‐NES_pCDNA3 which was a gift from Alice Ting (Addgene plasmid # 107169; http://n2t.net/addgene:107169; RRID:Addgene_107169) [64]. For transfection, CD9^−/−^ cells in Opti‐MEM media (Gibco) were treated with 0.5 μg of DNA and Lipofectamine 3000 for 24 h at 37 °C. Transduction was checked by protein electrophoresis and western blotting, while cells were immediately treated with biotin for analysis of nonspecific cytosolic biotinylation.

### Confocal microscopy

Cell membrane proteins were visualised as described previously [7]. Cells were grown overnight on glass coverslips and fixed with 4% paraformaldehyde. Coverslips were washed, blocked in 10% goat serum (Merck) and treated with anti‐CD9, anti‐CD44, anti‐CD46 or anti‐CD147 antibodies followed by goat anti‐mouse AlexFluor488 and anti‐rabbit AlexaFluor568 conjugated antibody. Cells were washed and stained with DAPI (4′,6‐diamidino‐2‐phenylindole) to visualise cell nuclei. Coverslips were mounted with Vectashield mounting medium with DAPI (Vector Labs, Newark, CA, USA), allowing visualisation on a Zeiss LSM 980 Airyscan microscope. Colocalisation coefficients were calculated using the JACoP plugin for imagej.

### Calcein AM adhesion assay

Black tissue culture‐treated 96‐well plates were coated with 75 μg/mL fibronectin (Merck) for 3 h. Wells were washed with PBS and seeded with 2 × 10^4^ cells for one hour or 24 h. Cells were washed twice with PBS and stained with 0.01 μm Calcein AM for one hour. Fibronectin‐coated wells without cells were used as a blank control. Fluorescence was read using the FLUOstar‐OPTIMA plate reader (488/520 nm). Cell adherence was calculated as a percentage of adherence by WT cells, set at 100%.

### Infection assays

Infection assays were carried out as previously described [4]. Cells were seeded onto 96‐well plates and cultured overnight. To reduce nonspecific binding, wells were blocked with 5% bovine serum albumin (BSA; Merck). Cells were treated with peptide for 60 min before infection with either MC58 or SH1000 at a multiplicity of infection (MOI) of 50 for 60 min at 37 °C with 5% CO_2_. Cells were washed and lysed with 2% saponin (Merck) for 30 min. Serial dilutions of lysates were plated onto BHI with 10% Levinthal's solution or LB agar plates and allowed to grow overnight. The number of bacteria bound to BSA‐blocked empty wells was subtracted from adherent and internalised bacteria. Bacterial adherence to peptide‐treated cells was calculated as a percentage of bacterial adherence to untreated cells, set at 100%.

To analyse the CD9 interactome during infection, CD9^−/−^ or CD9^−/−^ cells transfected with CD9‐TurboID were grown overnight to confluency in 100 mm tissue culture dishes. Dishes were blocked for 60 min with 5% BSA (Merck) before infection with MC58 or SH1000 at an MOI of 50. Uninfected and infected cells were treated with 50 μm biotin at the point of infection. Infection was allowed to proceed for 30, 60 or 240min before cells were washed, harvested with cell dissociation solution (Thermo Fisher Scientific) and lysed in cold RIPA buffer.

To analyse the number of adherent and internalised bacteria by microscopy, 1.5 × 10^5^ cells were seeded onto glass coverslips and cultured overnight. Infection assays were carried out as above and cells were washed with PBS after infection to remove nonadherent bacteria. Cells were fixed using a methanol:acetic acid (3:1) solution for 5 min before washing and staining with 10% Giemsa for 20 min. Coverslips were mounted using DPX mounting medium and viewed under a light microscope. Various fields of view were analysed to enumerate one hundred cells which were scored for the amount of infected cells and the total number of adherent bacteria.

### Biotin pull‐down

Biotinylated proteins were extracted from cell lysates using Dynabeads™ M‐280 streptavidin magnetic beads (Thermo Fisher Scientific). 50 μL of beads were washed in cold RIPA buffer without protease inhibitors before cell lysates were added and incubated with end over end rotation, overnight at 4 °C. Supernatants were removed and consecutively washed with the following; (i) 2% SDS/50mm Tris pH7.4, (ii) cold RIPA buffer, (iii) 2 m urea/50 mm ammonium bicarbonate, (iv) 50 mm ammonium bicarbonate.

### Mass spectrometry

Mass spectrometry experiments were carried out as described previously with minor modifications [65]. Briefly, streptavidin purifications were reduced and alkylated using 5 mm tri‐(2‐carboxyethyl) phosphine hydrochloride (TCEP) and 10 mm iodoacetamide, respectively. 2 μg trypsin (MS Grade; Pierce, Thermo Fisher Scientific) was added to each sample for on‐bead digestion and incubated for 3 h at 37 °C. Peptides were acidified through the addition of trifluoroacetic acid (TFA), desalted using C18 columns (Pierce, Thermo Fisher Scientific), and eluted peptides were dried using a vacuum concentrator (Eppendorf) at 45 °C. Peptides were resuspended in 12 μL 0.5% formic acid, and 5 μL of each sample was analysed by nanoflow LC–MS/MS using an Orbitrap Exploris 480 (Thermo Fisher Scientific) mass spectrometer with an EASY‐Spray source coupled to a Vanquish LC System (Thermo Fisher Scientific). Peptides were desalted online using a Pepmap Neo C18 nano trap column, 300 μm I.D.X 5 mm (Thermo Fisher Scientific) and then separated using an EASY‐Spray column, 50 cm × 75 μm ID, PepMap Neo C18, 2 μm particles, 10 Å pore size (Thermo Fisher Scientific). The 90 min gradient was used, starting from 3% to 20% buffer B (0.5% formic acid in 80% acetonitrile) for 60 min, then ramping up to 35% buffer B for 15 min, then up to 99% buffer B for 1 min, and maintaining at 99% buffer B for 9 min. The Orbitrap Exploris was operated in positive mode with a DDA cycle time of 2 s. MS1 spectra were acquired at a resolution of 120 000 at m/z 200, with a scan range (m/z) 375–1200, the standard AGC target. The most abundant multiply charged (2+ and higher) ions in a given chromatographic window were subjected to HCD fragmentation with a collision energy of 30% and dynamic exclusion set to automatic. The MS2 AGC target was set to standard, and MS2 spectra were measured with a resolution setting of 30 000 at m/z 200.

### Proteomic data analysis

As described previously [65], all raw mass spectrometry data were processed with MaxQuant version 1.6.10.43. Data were searched against a human (July 2022), *S. aureus* NCTC 8325 (May 2023) and *N. meningitidis* MC58 (July 2023) UniProt sequence database using the following search parameters: digestion set to Trypsin/P with a maximum of 2 missed cleavages, methionine oxidation and N‐terminal protein acetylation as variable modifications, cysteine carbamidomethylation as a fixed modification, match between runs enabled with a match time window of 0.7 min and a 20 min alignment time window, label‐free quantification enabled with a minimum ratio count of 2, minimum number of neighbours of 3 and an average number of neighbours of 6. A first search precursor tolerance of 20 ppm and a main search precursor tolerance of 4.5 ppm were used for FTMS scans and a 0.5 Da tolerance for ITMS scans. A protein FDR of 0.01 and a peptide FDR of 0.01 were used for identification level cut‐offs. MaxQuant output was loaded into Perseus version 1.6.10.50, and the matrix was filtered to remove all proteins that were potential contaminants, only identified by site and reverse sequences. LFQ intensities were log_2_(x) transformed, and data were filtered to retain proteins with a minimum of three valid LFQ intensities in one group. Subsequently, data were visualised using multi‐scatter plots and Pearson's correlation analysis. Data were normalised by subtracting column medians, and missing values were imputed from the normal distribution with a width of 0.3 and downshift of 1.8. To identify quantitatively enriched proteins between groups, two‐sided Student's *t*‐tests were performed with a permutation‐based FDR of 0.01 with an s0 = 2. Significantly enriched proteins were compared between uninfected and infected cells using two‐sided Student's *t*‐tests with a permutation‐based FDR of 0.05 and s0 = 0.1. The mass spectrometry proteomics data have been deposited to the ProteomeXchange Consortium via the PRIDE [66] partner repository with the dataset identifier PXD058283 and PXD067302. Data were exported into an Excel file and input into GraphPad Prism to create the figures and plots presented. Gene ontology and pathway analysis were performed using SubcellulaRVis [67] and WebGestalt [68].

### Co‐immunoprecipitation

Whole cell lysates from CD9^−/−^ cells transfected with the CD9:TurboID fusion protein or eGFP were prepared in RIPA buffer with a protease inhibitor cocktail (Roche). Protein‐G magnetic beads (Dynabeads, Thermo Fisher Scientific) were primed with 4 μg of a rabbit anti‐V5 monoclonal antibody on a rotator for 10 min at room temperature. V5‐tagged protein was pulled down by incubating lysates with labelled beads for 60 min on a rotator at 4 °C. Beads were washed in PBS with Triton X‐100 (0.2%) three times. Proteins were eluted by boiling in RIPA buffer and separated by SDS‐PAGE. Resulting blots were probed with rabbit anti‐V5, mouse anti‐CD44 or mouse anti‐CD147 antibodies (1:1000) and imaged with the appropriate secondary HRP‐conjugated antibody.

### siRNA knockdown

Target genes were knocked down using siGENOME SMARTpool human CD44 siRNA (M‐009999‐03), human CD46 siRNA (M‐004570‐00) or human CD147 siRNA (M‐010737‐01). Nontargeting siRNA pool (D‐001206‐13; Horizon Discovery Ltd., Cambridge, UK) was used as an appropriate control. 1.5 × 10^5^ WT or CD9^−/−^ cells were seeded onto 6‐well plates and cultured overnight. 5 μL of siRNA was mixed with 245 μL of serum‐free media, while 3 μL of Dharmafect 1 (Horizon Discovery) was added to 247 μL of serum‐free media. Both were left at room temperature for 5 min before mixing and incubated for a further 20 min before adding dropwise to cells. Cells were incubated for 48 h before harvesting with trypsin/EDTA and preparing for infection assays in 96‐well plates as described. Knockdown was confirmed by flow cytometry.

### Internalisation assays

Infection assays were performed as described above at 4 °C for one hour. Following infection, cells were washed with PBS to remove nonadherent bacteria before incubation at 37 °C for 30, 60 and 240 min to allow for internalisation of adherent bacteria. The number of internalised bacteria was also checked at *t* = 0. Cells were washed with PBS and treated with a kill solution of 200 μg/mL gentamicin and 20 μg/mL lysostaphin diluted in cell media for one hour. After killing the remaining external bacteria, cells were washed twice with PBS and lysed with 2% saponin to release internalised bacteria. Resulting lysates were serially diluted and colonies enumerated on chocolate agar.

### Statistical analyses

All analyses were performed within GraphPad Prism version 10.2.2 (GraphPad Software Inc., USA). Significance was established at *P* ≤ 0.05, and all data represents at least three independent experiments unless otherwise stated. Statistical considerations and specific analyses are described separately within each section. * specify significance to the untreated control unless otherwise specified; *P ≤ 0.05, **P ≤ 0.01, ***P ≤ 0.001.

## Author contributions

Conceptualisation, LRG and JGS; methodology, LRG, JGS, MOC, RHT, PAW and IFP; formal analysis, LRG, PAW and IFP; investigation, LRG, RHT, PAW and IFP; writing—original draft, LRG and IFP; writing—review and editing, LRG, PAW, IFP, RHT, JGS and MOC; visualisation, LRG; funding acquisition, LRG; supervision, LRG and JGS.

## Conflict of interest

All other authors declare that the research was conducted in the absence of any commercial or financial relationships that could be construed as a potential conflict of interest.

## Supporting information


**Data S1.** Proteins identified by proximity labelling mass spectrometry for untreated and infected cells. Excel file containing biotinylated proteins identified by mass spectrometry in A549 cells expressing a CD9:TurboID fusion protein. Significantly enriched proteins, deemed the CD9 interactome, are identified in comparison to A549 cells expressing a cytosolic TurboID at 30, 60 and 240 minutes. Data file also contains enriched proteins after infection with either *Neisseria meningitidis* or *Staphylococcus aureus* after 30, 60 and 240 min.
**Data S2**. Cell compartment and cellular pathway analysis of identified proteins. Data files contain analysis of significantly enriched proteins identified by mass spectrometry at 30, 60 and 240 min. Data files supplied by SubcellulaRVis demonstrates the cellular compartment analysis of the enriched proteins. Data files supplied by WebGestalt provide the KEGG pathway analysis of the significantly enriched proteins.

## Data Availability

The mass spectrometry proteomics data have been deposited to the ProteomeXchange Consortium with the dataset identifiers PXD058283 and PXD067302. Data S2 has been deposited to Dryad, with the unique DOI: https://doi.org/10.5061/dryad.m905qfvfc.
